# Prediction of Postoperative Speech Dysfunctions in Neurosurgery Based on Cortico-Cortical Evoked Potentials and Machine Learning Technology

**DOI:** 10.17691/stm2022.14.1.03

**Published:** 2022-01-28

**Authors:** T.A. Ishankulov, G.V. Danilov, D.I. Pitskhelauri, O.Yu. Titov, A.A. Ogurtsova, S.B. Buklina, E.V. Gulaev, T.A. Konakova, A.E. Bykanov

**Affiliations:** Engineer, Laboratory of Biomedical Informatics and Artificial Intelligence; N.N. Burdenko National Medical Research Center for Neurosurgery, Ministry of Health of the Russian Federation, 16, 4^th^ Tverskaya-Yamskaya St., Moscow, 125047, Russia;; Scientific Secretary, Head of the Laboratory of Biomedical Informatics and Artificial Intelligence; N.N. Burdenko National Medical Research Center for Neurosurgery, Ministry of Health of the Russian Federation, 16, 4^th^ Tverskaya-Yamskaya St., Moscow, 125047, Russia;; Professor, Head of the Department of Neurosurgery No.7; N.N. Burdenko National Medical Research Center for Neurosurgery, Ministry of Health of the Russian Federation, 16, 4^th^ Tverskaya-Yamskaya St., Moscow, 125047, Russia;; Clinical Resident, Department of Neurosurgery No.7; N.N. Burdenko National Medical Research Center for Neurosurgery, Ministry of Health of the Russian Federation, 16, 4^th^ Tverskaya-Yamskaya St., Moscow, 125047, Russia;; Neurophysiologist; N.N. Burdenko National Medical Research Center for Neurosurgery, Ministry of Health of the Russian Federation, 16, 4^th^ Tverskaya-Yamskaya St., Moscow, 125047, Russia;; Professor, Neuropsychologist; N.N. Burdenko National Medical Research Center for Neurosurgery, Ministry of Health of the Russian Federation, 16, 4^th^ Tverskaya-Yamskaya St., Moscow, 125047, Russia;; Neurophysiologist; National Medical Research Center for Traumatology and Orthopedics named after N.N. Priorov, Ministry of Health of the Russian Federation, 10 Priorova St., Moscow, 127299, Russia; PhD Student, Department of Radiology; N.N. Burdenko National Medical Research Center for Neurosurgery, Ministry of Health of the Russian Federation, 16, 4^th^ Tverskaya-Yamskaya St., Moscow, 125047, Russia;; Neurosurgeon, Researcher; N.N. Burdenko National Medical Research Center for Neurosurgery, Ministry of Health of the Russian Federation, 16, 4^th^ Tverskaya-Yamskaya St., Moscow, 125047, Russia;

**Keywords:** cortico-cortical evoked potentials, machine learning, artificial intelligence, neuro-oncology, glial tumors, speech function, connectome

## Abstract

Intraoperative recording of cortico-cortical evoked potentials (CCEPs) enables studying effective connections between various functional areas of the cerebral cortex. The fundamental possibility of postoperative speech dysfunction prediction in neurosurgery based on CCEP signal variations could serve as a basis to develop the criteria for the physiological permissibility of intracerebral tumors removal for maximum preservation of the patients’ quality of life.

**The aim of the study** was to test the possibility of predicting postoperative speech disorders in patients with glial brain tumors by using the CCEP data recorded intraoperatively before the stage of tumor resection.

**Materials and Methods:**

CCEP data were reported for 26 patients. To predict the deterioration of speech functions in the postoperative period, we used four options for presenting CCEP data and several machine learning models: a random forest of decision trees, logistic regression, and support vector machine method with different types of kernels: linear, radial, and polynomial. Twenty variants of models were trained: each in 300 experiments with resampling. A total of 6000 tests were performed in the study.

**Results:**

The prediction quality metrics for each model trained in 300 tests with resampling were averaged to eliminate the influence of “successful” and “unsuccessful” data grouping. The best result with F1-score = 0.638 was obtained by the support vector machine with a polynomial kernel. In most tests, a high sensitivity score was observed, and in the best model, it reached a value of 0.993; the specificity of the best model was 0.370.

**Conclusion:**

This pilot study demonstrated the possibility of predicting speech dysfunctions based on CCEP data taken before the main stage of glial tumors resection; the data were processed using traditional machine learning methods. The best model with high sensitivity turned out to be insufficiently specific. Further studies will be aimed at assessing the changes in CCEP during the operation and their relationship with the development of postoperative speech deficit.

## Introduction

One of the main tasks of modern brain science is to identify the structural and functional neural networks that maintain human cognitive functions. Identifying and preserving these networks during an operation is the most difficult and not completely solved problem of brain tumor neurosurgery. The analysis of intracerebral connections has become so significant that it is regarded as a new field of research called Brain Connectomics [1].

Connectomics considers the brain as a complex of elements (cortical regions, subcortical nuclei) united by three types of connectivity — structural, functional, and effective.

Effective connectivity is a directed flow of information between nerve structures [2, 3], which makes them work as a “signal source–signal receiver” system. Intravital examination of the effective connections is a challenging multidisciplinary problem, which is approached by intraoperative recording of cortico-cortical evoked potentials (CCEP) [4].

In the present study, to predict speech deterioration in the early postoperative period, we analyzed the CCEP recorded during surgery for brain glial tumor removal. The hypothesis tested by us was that the CCEP parameters measured before the main stage of surgical intervention could serve as predictors for worsening of the postoperative speech function. Confirmation of this hypothesis may become the basis for the development of criteria for the physiological feasibility of removing intracerebral tumors in order to maximize the patients’ quality of life.

## Materials and Methods

This prospective study included consecutive patients with intracerebral tumors located in the speech-dominant hemisphere, in close proximity to Broca’s and/or Wernicke’s speech areas, while the tumors’ medial part, according to preoperative MRI tractography, extended to the fibers of the arcuate fasciculus.

Before surgery, all patients were examined using a Signa HDxt 3.0T tomograph (GE Healthcare, USA). The MRI examination protocol included MRI scans run in the standard modes (T1-WI, T1 + C, 3D-T1-WI, T2-FLAIR, DWI), MRI tractography, and fMRI.

Before the surgery and 7 days after it, neurological and neuropsychological examinations were performed according to the Luria’s method. The preservation of speech function was assessed.

Microsurgical removal of the tumor was performed using craniotomy (the patient remained conscious) according to the protocol of monitored sedation. The study was conducted in accordance with the principles of the Helsinki Declaration (2013); informed consent was obtained from each patient.

### CCEP recording

Intraoperative recording of CCEP [5–7] was carried out using a 32-channel Neuro-IOM intraoperative monitoring system (Neurosoft LLC, Russia) and a pair of subdural electrode strips. The recorded CCEP data were processed by the original software developed by Neurosoft LLC as well; that guaranteed full compatibility between the recording systems and the data formats.

One of the two electrodes was placed in the frontal speech area (Broca’s area), the second was placed on the surface of the superior temporal gyrus in its posterior sections and on the supramarginal gyrus.

The CCEP data were recorded before and after the tumor resection, by averaging the evoked responses (30–50 stimuli per session) with an electrocorticogram analysis epoch of 300 ms, starting from the stimulus onset. To confirm the reproducibility of the response, at least two averaged curves were recorded each time.

Electrocorticography (ECoG) was performed with a quantization frequency of 20 kHz. Pass filters were set within 5–1000 Hz. Measurements from the subdural electrode were performed in the monopolar mode; a spiral subcutaneous electrode placed in the area of the contralateral mastoid process or in the frontal area was the reference.

Under conditions of bipolar montage, electrical stimulation of the cortex was performed using two adjacent contacts of the subdural electrode. The stimulation mode included single rectangular biphasic DC pulses with a duration of 300 μs and a frequency of 1 Hz. The stimulus intensity was raised gradually, starting from 2 mA, until the appearance of the paraphasic phenomena or epileptiform patterns on the ECoG. As a rule, the intensity range of 3–4 mA was the most commonly used.

### Data description.

CCEPs were obtained intraoperatively in 26 patients. In 14 patients, the CCEPs were examined before and after the main stage of surgical intervention, in the remaining 12 patients — before the main stage only. Each CCEP test was saved in a separate European Data Format (EDF) file that included ECoG waveform data in 8 or 16 channels. The number of tests for each patient was not limited — there were from 2 to 16 CCEP tests per patient.

**Figure 1:**
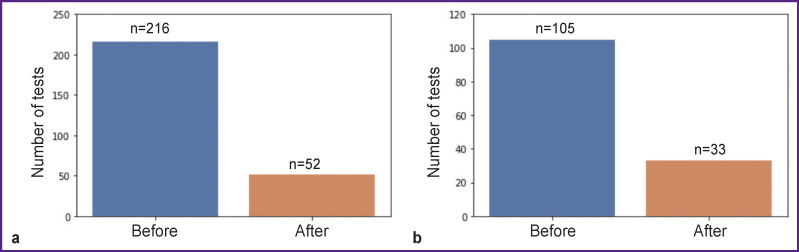
Number of pre- and post-surgical tests with CCEP recordings: (a) before the dataset screening by neurophysiologists; (b) after the dataset screening by neurophysiologists

The dataset obtained in this way after testing 26 patients included numerous files (n=268, 1 file for each test), of which 216 files contained CCEP data before surgery, and 52 files — after it (Figure 1). All the data obtained were analyzed independently by two neurophysiologists in order to assess the quality of ECoG recordings. Then, using the due software, the data were sorted into unsuitable and suitable for further analysis. After screening out low-quality records, 138 tests remained; of those, 105 related to the CCEP data obtained before surgery and 33 — after it.

**Figure 2:**
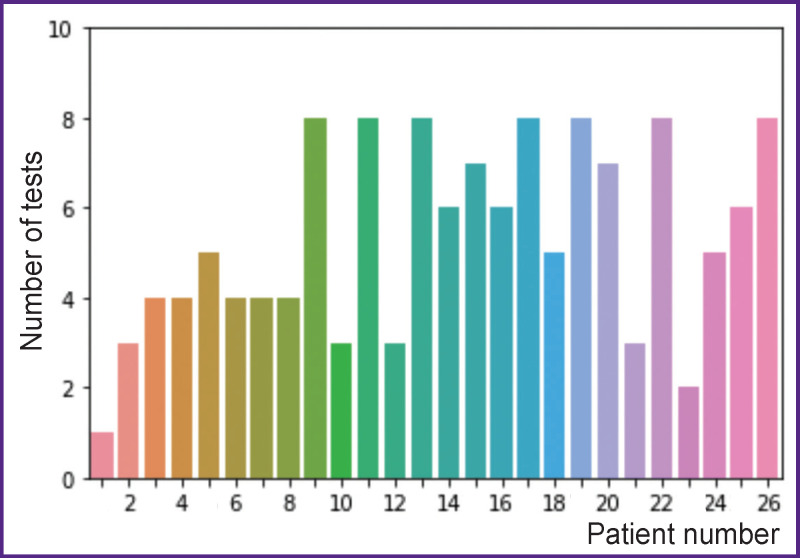
Number of tests in each patient

The number of tests conducted in each patient is shown in Figure 2. Due to the small number of complete data sets (before and after surgery), by now, our analysis is limited to preoperative CCEPs only (n=105).

### Signal preprocessing

To start the CCEP recording procedure, a single stimulus of 300 μs was applied. The duration of signal recording after stimulation was 300 ms. The analog signal was converted into the digital one at a sampling rate of 25,000 samples per second, therefore, each signal consisted of 7500 discrete values.

An individual patient test included ECoG data obtained via 8 or 16 channels. For all tests, the data were converted to the identical format. Most of the recorded curves appeared in the form of impulse noises with signal oscillations at very low amplitudes; therefore, it was decided to analyze the data obtained from the channel with the highest amplitude of signal oscillations.

**Figure 3:**
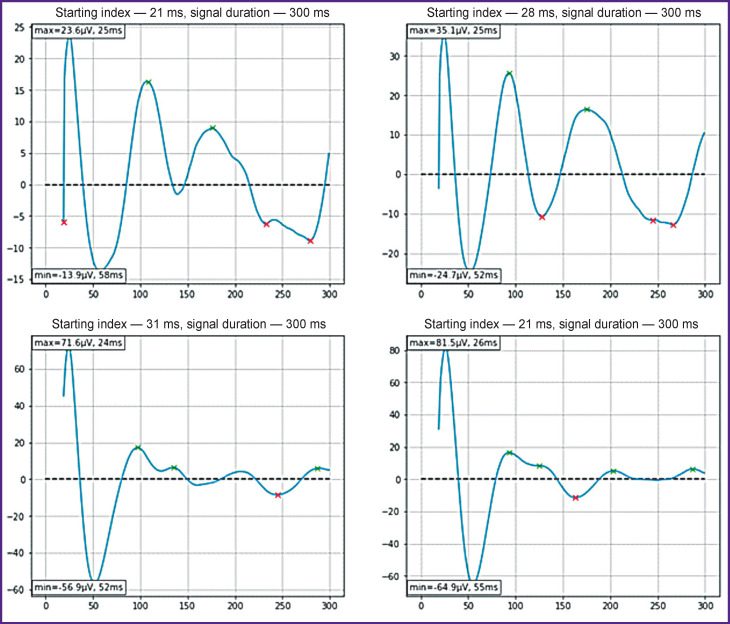
Examples of averaged and smoothed signals The time scale is shown on the abscissa axis, the signal parameters — on the ordinate axis. On each graph, the signal starting index, as well as the minimum and maximum values are indicated. The first 19 ms of the signal duration was used to calculate the moving average with a window of 20 ms; therefore, the first signal value was noted at the 20th ms. Then the index with the first value was shifted to the right (for 1 ms, at least) to remove the artifact

Before determining the channel with the highest signal amplitude, the data were averaged and smoothed. A vector of 7500 values was divided into 300 equal parts, 25 values each. The means of every 25 values formed a new vector consisting of 300 means. The vectors obtained for each patient had sharp jumps in values (spikes) throughout the entire signal duration. To smooth the signal, the moving average method with a window of n=20 was applied. Thus, the first 20 ms of the signal was used to calculate the first value of the smoothed signal. It significantly reduced the number of recorded artifacts. If the smoothed values had artifacts, they were automatically eliminated through the comparison with the amplitude of the rest of the signal multiplied by 1.25. If this value was exceeded, the starting index was shifted by a maximum of 10 values to the right. In addition, the signal starting index was always increased by 1 ms, even if no artifact was observed, in order to exclude the data from the first millisecond of the signal. Examples of averaged and smoothed signals are shown in Figure 3.

The new averaged and smoothed vectors were compared with each other in terms of the oscillation amplitude, and then the signal with the highest amplitude was selected. The selected signals had different durations due to the shift of the starting index. Time series analysis and indicators independent on signal duration were then applied.

### Generation of descriptive features of the signals

The initial descriptive features of the signals were determined after consultation with an expert physician. These features (commonly used by neurophysiologists to characterize CCEP records) included signal amplitude, wave type, and latency to the peak (positive or negative) value [5, 8, 9]. For each patient, the expert doctor provided a description of speech dysfunctions before and after surgery.

For each individual test, the maximum signal amplitude from one of 8 or 16 channels, the average amplitude for all channels, and the minimum signal amplitude from one of 8 or 16 channels were calculated. Further calculations were carried out for the channel with the highest amplitude.

For all features of the signal, the mean value was calculated and used as an additional feature. This mean value could be a positive or negative number. We assumed that this indicator would be useful for the quality of classification.

Peak values (local extrema) were determined using the SciPy software package for Python 3.8.5. The built-in function calculated the local extrema with a minimum distance between peaks of 20 ms and a minimum peak height of 5 μV, which made it possible to identify extrema with a greater accuracy. In Figure 3, the green cross marks indicate the highs, and the red marks indicate the lows. Up to 2 maximum values and up to 2 minimum values were used for the analysis. The missing extremum values were restituted in two ways: with zeros or the mean values of the ratios of the first peak multiplied by the amplitude of the first peak to the amplitude, and for the second peak, the mean values of the ratios of the second peak to the first peak multiplied by the value of the first peak. Filling in the missing values with zeros resulted in a higher quality of classification.

Additionally, for each selected signal, an augmented Dickey–Fuller test was performed to determine the stationarity of the time series. The p-values obtained from the test were compared to the critical significance levels and used as a feature for the models. The data from the extended Dickey–Fuller test were not included in the final models; all the time series turned out to be non-stationary.

After computing the features, the MinMaxScaler normalization method from the sklearn package was applied to the data; this treatment converted the values to a new range from 0 to 1 and reduced the data dimensionality.

**Figure 4:**
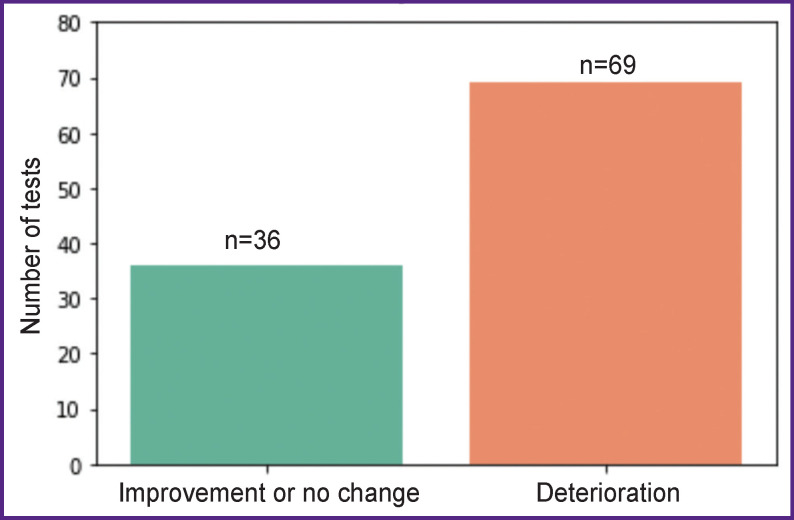
Changes in the patient’ speech function after surgery

In this study, we used a binary classification approach. To this end, we created a target variable based on the changes in the cumulative assessment of the patient’s speech dysfunctions after surgery ranging from 0 to 45 (0 is the norm). This binary target variable was set to 1 if the speech function worsened after surgery, and to 0 if the speech function improved or remained unchanged compared to the pre-surgery state (Figure 4).

### Mathematical models for the purpose of prediction

To predict the deterioration of the speech functions in the postoperative period, several machine learning models were used: a random forest (RF) of decision trees, logistic regression (LR), support vector machine (SVM) with different types of kernel: linear, radial, and polynomial (linear, Lin; radial basis function, RBF; polynomial, Poly).

Each test was performed after randomly sampled the data into training (80%) and testing (20%) subsets with stratification. The model was trained on the training set; then, 5-fold cross-validation (CV) was applied to evaluate the model’s quality before running it on the test set.

The data were divided into the testing and training subsets in two different ways. In the first method, the division was made over the entire range of the tests (n=105). In the second method, the division was carried out so that all the tests of a given patient fell into only one sample. Thus, if a patient underwent 6 tests, they would all fall into either the training or test set. This option for grouping the data by patient was based on the assumption that all tests of one patient were similar to each other and could led to the overfitting of the machine learning model.

In total, 4 variants of the input data were used:

1. data division by tests with filling in the missing values with mean values;
2. data division by tests with filling in the missing values with zeros;
3. data division by patients with filling in the missing values with mean values;
4. data division by patients with filling in the missing values with zeros.

For all four types of the input data, a few series of tests were carried out using each of the 5 machine learning models. Thus, 20 variants of the models were trained 300 times in these series of tests, which led to a total number of 6000 test runs.

## Results

We used standard metrics to evaluate the test results: accuracy on validation samples within the cross-validation (CV), specificity, and sensitivity (Spec and Sens, respectively), the proportion of correct classifier responses (Acc), precision and recall (Prec and Rec, respectively), F1-score and the area under the ROC-curve (area under curve, AUC).

The results for each series of 300 tests were averaged over all metrics to eliminate the influence of successful and unsuccessful data sets.

The results of the tests carried out with the first two variants of the input data are presented in Table 1. Comparison by the F-measure indicates that 3 out of 5 models showed an improvement in the quality of classification upon filling in the missing values with zeros.

**Table 1:** Classification results obtained upon dividing the data by tests

Model	CV	Spec	Sens	Prec	Rec	Acc	F1-score	AUC
***Filling in missing values with the mean values***								
RF	0.743	**0.535**	0.880	0.756	**0.708**	0.765	0.713	**0.708**
LR	0.725	0.240	0.993	0.812	0.617	0.742	0.603	0.617
SVM (Lin)	0.706	0.220	0.983	0.726	0.602	0.729	0.579	0.602
SVM (RBF)	0.754	0.303	0.995	0.831	0.649	0.764	0.645	0.649
SVM (Poly)	**0.756**	0.300	**0.999**	**0.841**	0.650	**0.766**	0.645	0.650
***Filling in missing values with zeros***								
RF	0.751	**0.559**	0.877	0.761	**0.718**	0.771	**0.723**	**0.718**
LR	0.724	0.237	**0.996**	0.807	0.617	0.743	0.601	0.617
SVM (Lin)	0.704	0.226	0.977	0.728	0.602	0.727	0.582	0.602
SVM (RBF)	0.756	0.293	0.995	0.838	0.644	0.761	0.640	0.644
SVM (Poly)	**0.759**	0.348	0.992	**0.842**	0.670	**0.777**	0.671	0.670

With the 3rd and 4th variants of the input data, we used the division by patients — all tests of each individual patient fell into one of two samples: training or testing (Table 2).

**Table 2:** Classification results obtained upon dividing the data by patients

Model	CV	Spec	Sens	Prec	Rec	Acc	F1-score	AUC
***Filling in missing values with the mean values***								
RF	0.665	0.286	0.829	0.556	0.557	0.612	0.519	0.557
LR	0.700	0.155	0.971	0.559	0.563	0.649	0.492	0.563
SVM (Lin)	0.687	0.072	0.954	0.387	0.513	0.606	0.417	0.513
SVM (RBF)	0.736	0.290	0.973	0.615	0.631	0.702	0.579	0.631
SVM (Poly)	**0.751**	**0.297**	**0.985**	**0.618**	**0.641**	**0.708**	**0.587**	**0.641**
***Filling in missing values with zeros***											
RF	0.680	0.319	0.809	0.569	0.564	0.606	0.530	0.564
LR	0.687	0.168	0.965	0.555	0.566	0.649	0.500	0.566
SVM (Lin)	0.674	0.098	0.944	0.411	0.521	0.612	0.432	0.521
SVM (RBF)	0.730	0.324	0.973	0.649	0.649	0.716	0.604	0.649
SVM (Poly)	**0.747**	**0.370**	**0.993**	**0.683**	**0.681**	**0.747**	**0.638**	**0.681**

The F1-score comparison shows an improvement in the quality of classification for all 5 models when filling in the missing values with zeros.

Classification under the division by patients (see Table 2) produced significantly lower results as compared with the division by tests (see Table 1), which indicated the involvement of a model overlearning factor. The results in Table 1 were not considered for the final comparison with the results of other models.

The best result for the F1-score metric was 0.638 for the SVM (Poly) model when filling in the missing values with zeros.

In most tests, a high sensitivity index was observed, and for the best SVM model (Poly), it reached 0.993. Thus, the model made correct predictions in 99.3% of patients with speech deterioration after surgery. At the same time, only 37% of patients with improvement/preservation of speech functions were correctly identified by the model. Along with the fact that the identification of patients at risk of speech deterioration was a top priority of the study, attributing some of the patients with speech improvement to the risk zone reduced the overall accuracy of the algorithm.

## Discussion

Attempts to determine the response of the cortex (local cortical response) and other brain structures to stimulation by a single electrical stimulus have been made since the 1960s and predominantly involved animal studies [10–12].

In the late 80s and early 90s, the first reports on functional connections between the temporal and limbic lobes as discovered with the help of evoked electrical potentials were published [13, 14]. In these studies, the response induced by stimulating brain structures at a distance from the stimulus electrode was detected, which made it possible to study the distant parts of the brain and their interconnections.

At the beginning of the XXI century, independently of each other, at the Cleveland Clinic [15], the University of Iowa [16], and King’s College London [17], scientists continued to develop methods for stimulating the cerebral cortex with a single impulse, as well as methods for analyzing the resulting evoked potentials. These works revived scientific interest in studying the signals, which are most often referred to as “cortico-cortical evoked potentials” (CCEP) according to the term introduced by the scientists from Cleveland [15].

In the present paper, an algorithm for predicting the deterioration or improvement/absence of changes in speech functions in the postoperative period was addressed using machine learning algorithms. According to the available literature, this is the first study in which machine learning methods were used to predict changes in the speech functions based on CCEP data.

According to the few published reports, researchers measured such CCEP parameters as the amplitude of signal oscillations and the latency to the signal peak [5, 8, 9, 18, 19]. In addition to these parameters, we calculated the average value over all the signal amplitudes, the latency to the local signal peaks (local extremum), and their absolute values in microvolts.

Signal preprocessing and transformation methods can be extended to add new features to the models, as well as to create new models based on these approaches. In the future, it is advisable to consider the methods of singular spectral analysis [20, 21], the use of wavelet transforms [22], Hilbert–Huang transforms [23], and other methods of working with time series [24, 25].

The limitations of this study include the relatively small sample size (n=26) and the insufficient number of patients whose post-surgical CCEP curves were recorded. Increasing the number of such patients may result in a higher quality of data classification.

Our approach to the task of classification was based on predicting a binary target variable: i.e. either deterioration or improvement in speech functions. This made it possible to regroup the data pool with a lower imbalance (see Figure 4) compared to dividing the target variable into several categories by the degree of speech impairment (in the latter case, there was a pronounced imbalance between the classes). Upon increasing the number of patients with different grades of speech dysfunctions, it will become possible to classify the cases by severity of the disorders.

In our future work, we plan to test new methods for predicting speech disorders, add new descriptive features to the existing models, as well as develop new machine learning models, including the ensemble ones.

## Conclusion

This pilot study demonstrated the ability to predict speech dysfunction developing in patients after brain surgery; the method is based on measuring cortico-cortical evoked potentials followed by data processing with the help of machine learning technology. Early detection of the speech dysfunction precursors according to the CCEP data can improve the results of surgical treatment in this functionally important area.

## References

[1] Sporns O (2011). The human connectome: a complex network.. Ann N Y Acad Sci.

[2] Leisman G., Moustafa A.A., Shafir T (2016). Thinking, walking, talking: integratory motor and cognitive brain function.. Front Public Health.

[3] Kunieda T., Yamao Y., Kikuchi T., Matsumoto R (2015). New approach for exploring cerebral functional connectivity: review of cortico-cortical evoked potential.. Neurol Med Chir (Tokyo).

[4] Matsumoto R., Nair D.R., LaPresto E., Bingaman W., Shibasaki H., Lüders H.O. (2007). Functional connectivity in human cortical motor system: a cortico-cortical evoked potential study.. Brain.

[5] Bykanov A.E., Pitskhelauri D.I., Titov O.Y., Lin M.C., Gulaev E.V., Ogurtsova A.A., Maryashev S.A., Zhukov V.Y., Buklina S.B., Lubnin A.Y., Beshplav S.T., Konakova T.A., Pronin I.N. (2020). Broca’s area intraoperative mapping with cortico-cortical evoked potentials.. Voprosy neirokhirurgii imeni N.N. Burdenko.

[6] Yamao Y., Matsumoto R., Kikuchi T., Yoshida K., Kunieda T., Miyamoto S (2021). Intraoperative brain mapping by cortico-cortical evoked potential.. Front Hum Neurosci.

[7] Tamura Y., Ogawa H., Kapeller C., Prueckl R., Takeuchi F., Anei R., Ritaccio A., Guger C., Kamada K (2016). Passive language mapping combining real-time oscillation analysis with cortico-cortical evoked potentials for awake craniotomy.. J Neurosurg.

[8] Saito T., Tamura M., Muragaki Y., Maruyama T., Kubota Y., Fukuchi S., Nitta M., Chernov M., Okamoto S., Sugiyama K., Kurisu K., Sakai K.L., Okada Y., Iseki H (2014). Intraoperative cortico-cortical evoked potentials for the evaluation of language function during brain tumor resection: initial experience with 13 cases.. J Neurosurg.

[9] Kubota Y., Enatsu R., Gonzalez-Martinez J., Bulacio J., Mosher J., Burgess R.C., Nair D.R (2013). In vivo human hippocampal cingulate connectivity: a corticocortical evoked potentials (CCEPs) study.. Clin Neurophysiol.

[10] Tielen A.M., Lopes da Silva F.H., Mollevanger W.J. (1981). Differential conduction velocities in perforant path fibres in guinea pig.. Exp Brain Res.

[11] Andersen P., Holmqvist B., Voorhoeve P.E (1966). Excitatory synapses on hippocampal apical dendrites activated by entorhinal stimulation.. Acta Physiol Scand.

[12] Gloor P., Vera C.L., Sperti L (1964). Electrophysiological studies of hippocampal neurons. III. Responses of hippocampal neurons to repetitive perforant path volleys.. Electroencephalogr Clin Neurophysiol.

[13] Wilson C.L., Isokawa M., Babb T.L., Crandall P.H (1990). Functional connections in the human temporal lobe. I. Analysis of limbic system pathways using neuronal responses evoked by electrical stimulation.. Exp Brain Res.

[14] Rutecki P.A., Grossman R.G., Armstrong D., Irish-Loewen S (1989). Electrophysiological connections between the hippocampus and entorhinal cortex in patients with complex partial seizures.. J Neurosurg.

[15] Matsumoto R., Nair D.R., LaPresto E., Najm I., Bingaman W., Shibasaki H., Lüders H.O. (2004). Functional connectivity in the human language system: a cortico-cortical evoked potential study.. Brain.

[16] Howard M.A., Volkov I.O., Mirsky R., Garell P.C., Noh M.D., Granner M., Damasio H., Steinschneider M., Reale R.A., Hind J.E., Brugge J.F (2000). Auditory cortex on the human posterior superior temporal gyrus.. J Comp Neurol.

[17] Valentín A., Anderson M., Alarcón G., Seoane J.J., Selway R., Binnie C.D., Polkey C.E. (2002). Responses to single pulse electrical stimulation identify epileptogenesis in the human brain in vivo.. Brain.

[18] Conner C.R., Ellmore T.M., DiSano M.A., Pieters T.A., Potter A.W., Tandon N (2011). Anatomic and electro-physiologic connectivity of the language system: a combined DTI-CCEP study.. Comput Biol Med.

[19] Silverstein B.H., Asano E., Sugiura A., Sonoda M., Lee M.H., Jeong J.W (2020). Dynamic tractography: integrating cortico-cortical evoked potentials and diffusion imaging.. Neuroimage.

[20] Mercier M., Bickel S., Megevand P., Groppe D., Mehta A (2015). Intracranial recording: a glimpse on white-grey matter differences.. Epilepsy Curr.

[21] Golyandina N (2020). Particularities and commonalities of singular spectrum analysis as a method of time series analysis and signal processing.. Wiley Interdiscip Rev Comput Stat.

[22] Torrence C., Compo G.P (1998). A practical guide to wavelet analysis.. Bull Am Meteorol Soc.

[23] Huang C.C., Chang C.S., Hsin Y.L (2015). Time–frequency spectral analysis of cortico-cortical evoked potentials by means of Hilbert–Huang transform.. Brain Stimul.

[24] Prime D., Woolfe M., Rowlands D., O’Keefe S., Dionisio S (2020). Comparing connectivity metrics in corticocortical evoked potentials using synthetic cortical response patterns.. J Neurosci Methods.

[25] Christ M., Braun N., Neuffer J., Kempa-Liehr A.W (2018). Time Series FeatuRe Extraction on basis of Scalable Hypothesis tests (tsfresh — a Python package).. Neurocomputing.

